# Cerebellar Hypoplasia in Mice Lacking Selenoprotein Biosynthesis in Neurons

**DOI:** 10.1007/s12011-014-9920-z

**Published:** 2014-03-06

**Authors:** Eva K. Wirth, B. Suman Bharathi, Dolph Hatfield, Marcus Conrad, Markus Brielmeier, Ulrich Schweizer

**Affiliations:** 1 Institut für Experimentelle Endokrinologie, Charité-Universitätsmedizin Berlin, Augustenburger Platz 1, 13353 Berlin, Germany; 2 Molecular Biology of Selenium, Mouse Cancer Genetics Program, Center for Cancer Research, National Institutes of Health, Bethesda, MD 20892 USA; 3 Helmholtz Zentrum München, Institute of Developmental Genetics, Ingolstädter Landstr. 1, 85764 Neuherberg, Munich Germany; 4 Abteilung für Vergleichende Medizin, Helmholtz Zentrum München, Ingolstädter Landstrasse 1, 85764 Neuherberg, Germany; 5 Institut für Biochemie und Molekularbiologie, Rheinische Friedrich-Wilhelms-Universität Bonn, Nussallee 11, 53115 Bonn, Germany

**Keywords:** Selenium, Brain, Gpx4, Cell death, Proliferation

## Abstract

Selenium exerts many, if not most, of its physiological functions as a selenocysteine moiety in proteins. Selenoproteins are involved in many biochemical processes including regulation of cellular redox state, calcium homeostasis, protein biosynthesis, and degradation. A neurodevelopmental syndrome called *progressive cerebello-cortical atrophy* (PCCA) is caused by mutations in the selenocysteine synthase gene, *SEPSECS*, demonstrating that selenoproteins are essential for human brain development. While we have shown that selenoproteins are required for correct hippocampal and cortical interneuron development, little is known about the functions of selenoproteins in the cerebellum. Therefore, we have abrogated neuronal selenoprotein biosynthesis by conditional deletion of the gene encoding selenocysteyl tRNA^[Ser]Sec^ (gene symbol *Trsp*). Enzymatic activity of cellular glutathione peroxidase and cytosolic thioredoxin reductase is reduced in cerebellar extracts from *Trsp*-mutant mice. These mice grow slowly and fail to gain postural control or to coordinate their movements. Histological analysis reveals marked cerebellar hypoplasia, associated with Purkinje cell death and decreased granule cell proliferation. Purkinje cell death occurs along parasagittal stripes as observed in other models of Purkinje cell loss. Neuron-specific inactivation of glutathione peroxidase 4 (Gpx4) used the same Cre driver phenocopies tRNA^[Ser]Sec^ mutants in several aspects: cerebellar hypoplasia, stripe-like Purkinje cell loss, and reduced granule cell proliferation. Parvalbumin-expressing GABAergic interneurons (stellate and/or basket cells) are virtually absent in tRNA^[Ser]Sec^-mutant mice, while some remained in *Gpx4*-mutant mice. Our data show that selenoproteins are specifically required in postmitotic neurons of the developing cerebellum, thus providing a rational explanation for cerebellar hypoplasia as occurring in *PCCA* patients.

## Introduction

Selenoproteins are proteins, which specifically have the rare amino acid, selenocysteine (Sec), incorporated in response to UGA codons in their mRNAs. The mouse and human genomes contain 24 and 25 genes encoding selenoproteins [1]. The mouse serves as a good model organism for mammalian brain development, and manipulation of its genome allows for easy studies of the physiological roles of selenoproteins. Most selenoproteins are expressed in the brain, particularly in neurons of the olfactory bulb, cortex, hippocampus, and cerebellum [2]. While dietary selenium (Se) deficiency is not known to induce direct neurological disease or neurodevelopmental delay in mammals [3], inactivation of the plasma Se transport protein, selenoprotein P (SePP), does lead to a neurological disorder which is caused by reduced brain Se content and selenoenzyme activity [4, 5]. Similarly, inactivation of apolipoprotein E receptor 2 (ApoER2, Lrp8), which serves as a SePP receptor in the brain, mimics the phenotype of SePP-KO mice [6, 7].

Selenoprotein biosynthesis relies entirely on the presence of tRNA^[Ser]Sec^, because Sec is synthetized attached to its tRNA, and this tRNA is also required for Sec incorporation by the ribosome [8]. Previously, we took an advantage of a cell type-specific genetic deficiency model of tRNA^[Ser]Sec^ [9–11] to abolish the expression of all selenoproteins simultaneously. When tRNA^[Ser]Sec^ was specifically inactivated in neurons, cytosolic glutathione peroxidase (Gpx) activity was reduced by 50 % in the forebrain on postnatal day 9 (P9), and Western blot on P12 demonstrated significant reductions of Gpx1, Gpx4, SePH, and SePR proteins approaching the detection limits [12]. This corresponded with predominantly neuronal expression of many selenoproteins. An interesting observation was the specific developmental defect of a certain subgroup of GABAergic interneurons [12], while Se and vitamin E deficiency in vitro also affected principal, glutamatergic neurons [13]. Neuron-specific deletion of *Gpx4* reduced forebrain Gpx4 protein by at least 50 % and was sufficient to disrupt interneuron development and mimic most phenotypes observed through the loss of tRNA^[Ser]Sec^ [12, 14]. Neuron-specific inactivation of thioredoxin reductase 1 (Txnrd1) or Txnrd2 alone does not change the cerebellar development, but inactivation of Txnrd1 in neural precursors leads to cerebellar hypoplasia [15]. Recently, a syndrome called *progressive cerebello-cerebral atrophy* (PCCA) was described which is caused by mutations in the selenocysteine synthase (*SEPSECS*) gene [16, 17]. Among the phenotypes described in those affected children is a marked cerebellar hypoplasia. Here, we report on the cerebellar development of mice lacking tRNA^[Ser]Sec^ specifically in neurons and demonstrate that the lack of selenoprotein expression in cerebellar neurons specifically entails cerebellar hypoplasia.

## Methods

### Animals

Mice were maintained according to local regulations as described for the *SePP*-deficient mice generated in our laboratory [5]. All animal experiments were approved by the local authorities in Berlin and Munich. Conditional *Trsp* knockout mice (*Trsp* ^*fl/fl*^) have been described [9]. These mice were crossed with transgenic mice expressing Cre recombinase under control of the tubulinα1 promotor [18], yielding mice deficient in neuronal selenoprotein biosynthesis, *T*α*1-Cre*/*Trsp* ^*fl/fl*^. Mutant mice and littermate controls were analyzed between P3 (not shown, no defects observed) and P15 (histology).

### Enzyme Assays

Cerebella were freshly dissected from postnatal mice and immediately frozen on dry ice. Brain tissue was powdered under liquid nitrogen using a dismembrator (Braun Melsungen), and aliquots were homogenized in buffer as described [5]. Activities of selenoenzymes Gpx and Txnrd were measured in cytosolic cerebellar homogenates as described with tBuOOH and bisdithionitrobenzene (DTNB) as substrates, respectively [5].

### Immunohistochemistry

Brains from mouse pups were immediately fixed after dissection in 4 % paraformaldehyde in 0.1 M phosphate buffer (PB), pH 7.4, or the mice were perfused transcardially with the same fixative after a washing step with 0.1 M PB. After postfixation at 4 °C over night, brains were cryoprotected in 30 % sucrose in 0.1 M PB for 2 days and frozen at −80 °C. Sections were cut on a cryostat at 20–50 μm. Free floating sections were stained with the indicated antibodies at dilutions of 1:1,000–1:5,000 at 4 °C over night. Polyclonal rabbit α-calbindin, monoclonal mouse α-calbindin, and rabbit α-parvalbumin antibodies were from Swant, Bellinzona, Switzerland, guinea pig monoclonal α-GLAST was from Chemicon, and mouse monoclonal α-glial fibrillary acidic protein (GFAP) antibody was from Sigma. Pre-absorbed secondary Cy2- and Cy3-conjugated antibodies were from Jackson ImmunoResearch. Horseradish peroxidase and diaminobezidine or NovaRed substrate were used in conjunction with the Vectastain ABC kit (Vector, Burlingame, VT). Whole-mount immunohistochemistry was performed as described [19]. Photomicrographs were taken using a Zeiss Axioskop II equipped with AxioCam MRc and operated with AxioVision software. Other photomicrographs were taken using a Leica confocal microscope.

### Terminal UTP-Nick End Labeling and Phosphorylated Histone H3 Assay

Terminal UTP-nick end labeling (TUNEL) immunohistochemistry was performed on serial 5-μm paraffin sections as described [20]. Using AxioVision software, TUNEL + cells were counted on at least 10 fields (0.35 mm^2^) for each genotype. Phosphorylated histone H3 (pH3) + cells were counted in serial sections and the numbers expressed per 1-mm external germinal layer (egl). For each measurement, at least 10 sections from at least three animals per genotype were evaluated. Antibodies against phosphorylated (Ser10) histone H3 were from Cell Signalling.

### Nissl Staining

Cresyl violet staining was performed on frozen 20-μm sections as described recently [21] and combined with α-calbindin immunostaining.

## Results

### Neuron-Specific Disruption of Selenoprotein Biosynthesis Reduces Selenoenzyme Activity

**Fig. 1:**
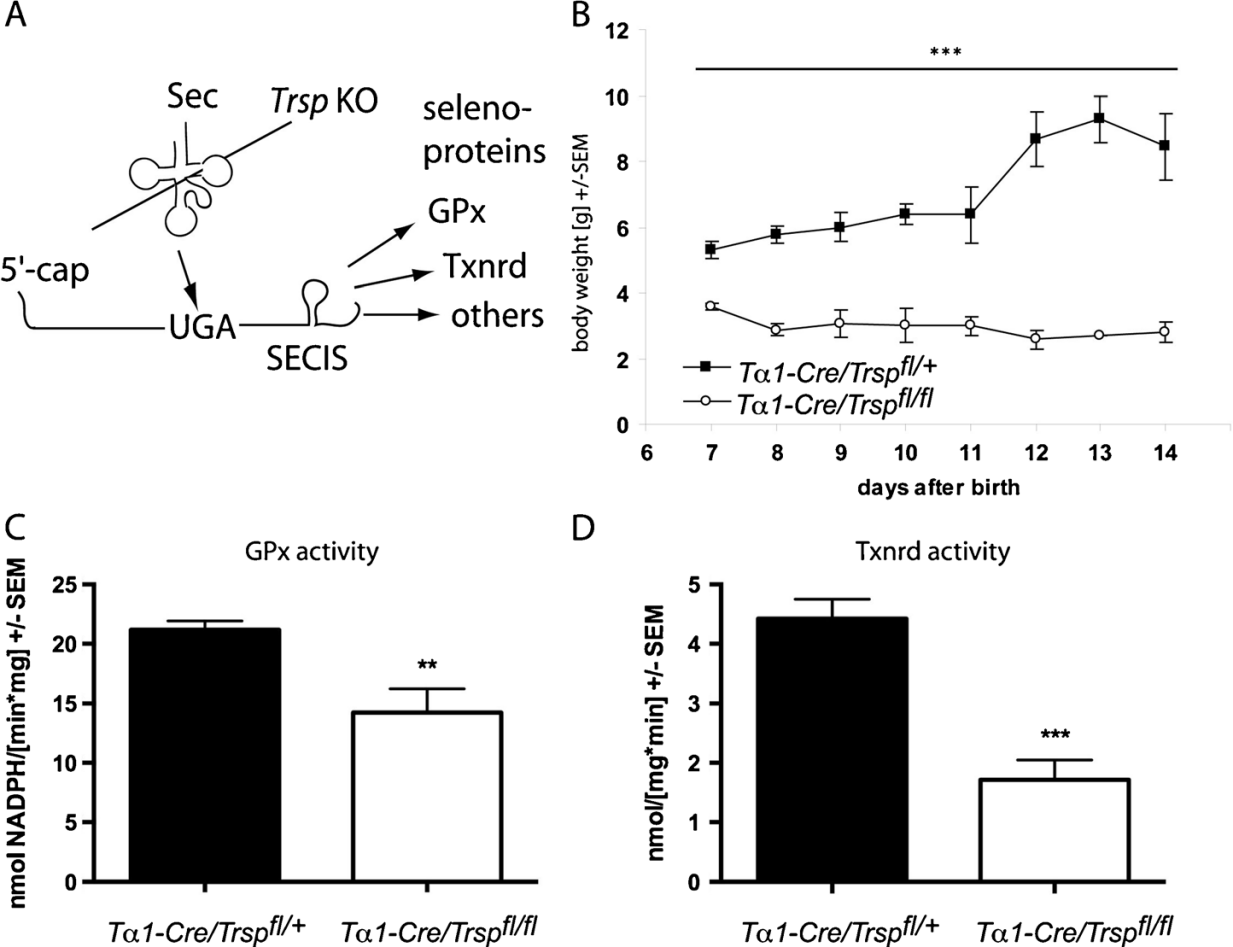
Neuron-specific inactivation of selenoprotein expression. **a** Schematic drawing shows dependence of selenoprotein synthesis on selenocysteine tRNA. **b** Body weight in postnatal *T*α*1-Cre*/*Trsp* ^*fl/fl*^ mice and controls. **c** Cytosolic glutathione peroxidase activity was determined with *tert*-butyl hydroperoxide as a substrate in P9 cerebella. **d** Cytosolic thioredoxin reductase activity was determined with the DTNB assay in P9 cerebella. *n* = 5–7, **p < 0.01; ***p < 0.001, Student’s *t* test

Selenoprotein expression was specifically abrogated in neurons by conditional inactivation of tRNA^[Ser]Sec^ (*Trsp*) based on neuron-specific expression of Cre recombinase (Fig. 1a). Neuron-specific *Trsp*-knockout mice (*T*α*1-Cre*/*Trsp* ^*fl/fl*^) failed to gain weight after the first week of life and rarely survived beyond P12 (Fig. 1b). Since the knockout animals did not gain postural control, we suspected a cerebellar defect and determined selenoprotein activities in cytosolic extracts of cerebella on P9. Glutathione peroxidase activity was reduced by 30 % (*p* < 0.01, Student’s *t* test; Fig. 1c), and cytosolic thioredoxin reductase was reduced by 60 % (*p* > 0.001; Student’s *t* test; Fig. 1d). Remaining selenoenzyme activities are likely derived from nonneuronal cells like glia and endothelial cells, which are not targeted by our approach as previously observed in the cerebral cortex [12].

### Cerebellar Hypoplasia and Stripe-Like Loss of Purkinje Cells

**Fig. 2:**
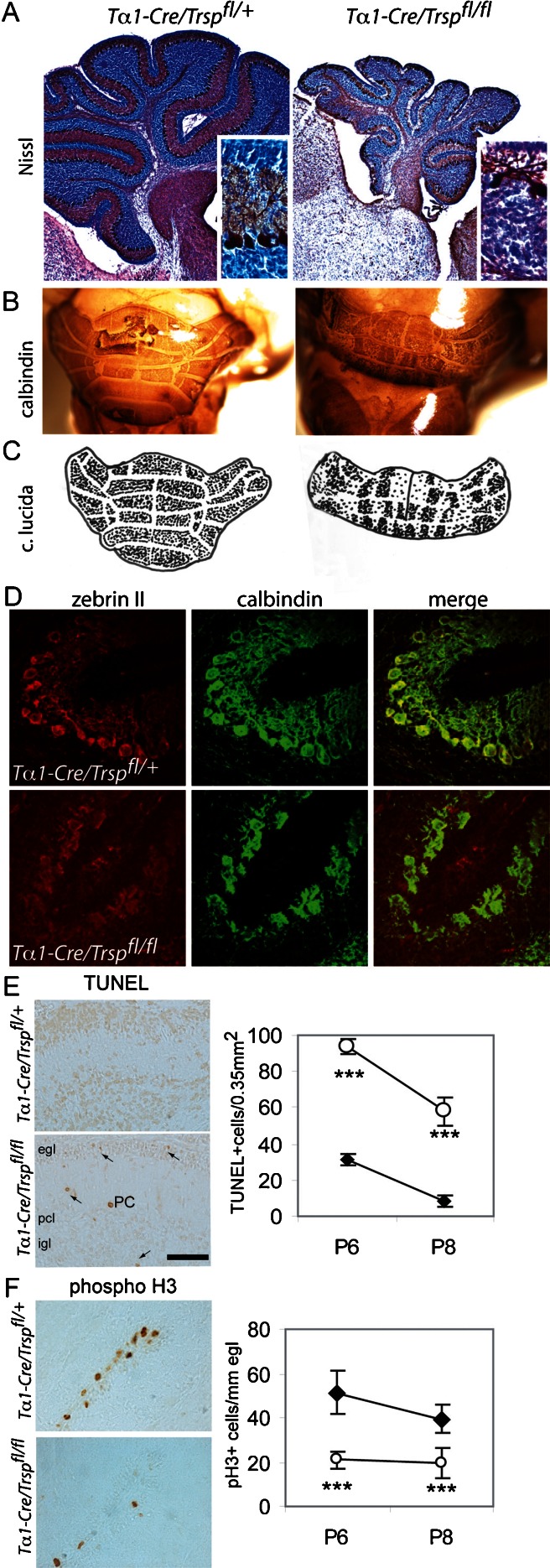
Cerebellar hypoplasia and Purkinje cell loss in mice lacking neuronal selenoprotein expression. *a* Nissl staining reveals marked cerebellar hypoplasia and immature foliation pattern in *T*α*1-Cre*/*Trsp* ^*fl/fl*^ mice on P12. The Purkinje cell layer is partially disrupted. *Insets* indicate stunted Purkinje cell dendrites and reduced external germinal layer (egl) thickness in *T*α*1-Cre*/*Trsp* ^*fl/fl*^ mice. α-Calbindin brown. *b* Whole-mount immunohistochemistry α-calbindin reveals stripe-like Purkinje cell loss on P12. *c* Camera lucida representation of Purkinje cells. *d* Co-expression of zebrin II (*red*) and calbindin (*green*) on P11. *e Left panel* TUNEL staining shows apoptosis on P8. Cell death (*arrows*) occurs in the egl, during migration in the molecular layer, and in the internal granule cell layer (igl). Occasionally, TUNEL + Purkinje cells (PC) are identified based on the large size of their nuclei. *Right panel* quantification of TUNEL + cells per 0.35 mm^2^ on P6 and P8. *Diamonds* indicate wild type and *open circles* mutant. *f Left panel* immunostaining for phosphorylated histone H3 (*phospho H3+*) as an indicator of proliferation. Mitosis in egl is clearly reduced in *T*α*1-Cre*/*Trsp* ^*fl/fl*^ cerebellum on P8. *Right panel* quantification of pH3+ cells per 1-mm egl. *Diamonds* indicate wild type and *open circles* mutant. ***p < 0.001, Student’s *t* test. *Scale bar* = 50 μm

The most striking anatomical phenotype of *T*α*1-Cre*/*Trsp* ^*fl/fl*^ mice was marked as cerebellar hypoplasia associated with immature foliation (Fig. 2(a)). Staining for Purkinje cells with antibodies against either calbindin or parvalbumin showed a significant loss of Purkinje cells in most folia on P7 and later stages. No Purkinje cell loss or size difference was apparent on P3 (not shown). Purkinje cell dendrites were clearly underdeveloped in *T*α*1-Cre*/*Trsp* ^*fl/fl*^ cerebella on P12 (Fig. 2(a), insets), and occasionally, Purkinje cells were ectopically distributed within deep layers of the white matter. We noted that the degree of Purkinje cell loss varied among parasagittal sections from the same animal. Since Purkinje cell death is known to occur along parasagittal stripes in several models of Purkinje cell degeneration [22], we performed whole-mount immunohistochemistry using calbindin as a marker for Purkinje cells. Calbindin staining revealed a stripe-like pattern of Purkinje cell loss (Fig. 2(b)) that was readily apparent in a *camera lucida* representation (Fig. 2(c)). In order to assess whether Purkinje cell loss coincided with the pattern of expression of zebrin II/aldolase C, we stained cerebellar sections with calbindin and zebrin II antibodies (Fig. 2(d)). In the primary fissure, surviving Purkinje cells were zebrin II + on P11.

### Cell Proliferation and Cell Death

TUNEL staining demonstrated that cell death was significantly increased in *T*α*1-Cre*/*Trsp* ^*fl/fl*^ cerebella on P6 and P8. Increased TUNEL labeling was not limited to the Purkinje cell layer but was also found in the external germinal layer and internal granule cell layer (Fig. 2(e)). The massive increase in size of the cerebellum during postnatal development is largely caused by the proliferation of granule cell precursors in the secondary neuroepithelium of the external germinal layer. In *T*α*1-Cre*/*Trsp* ^*fl/fl*^ mice, the external germinal layer was clearly reduced in thickness (Fig. 2(a), insets). In order to test whether granule cell proliferation was reduced in *T*α*1-Cre*/*Trsp* ^*fl/fl*^ cerebella, we stained serial cerebellar sections on P6 and P8 with antibodies against pH3. The number of proliferating granule cells in the external germinal layer was significantly reduced in *T*α*1-Cre*/*Trsp* ^*fl/fl*^ mice as compared to controls (Fig. 2(f)).

### Inactivation of Gpx4 Recapitulates the Cerebellar Phenotype

**Fig. 3:**
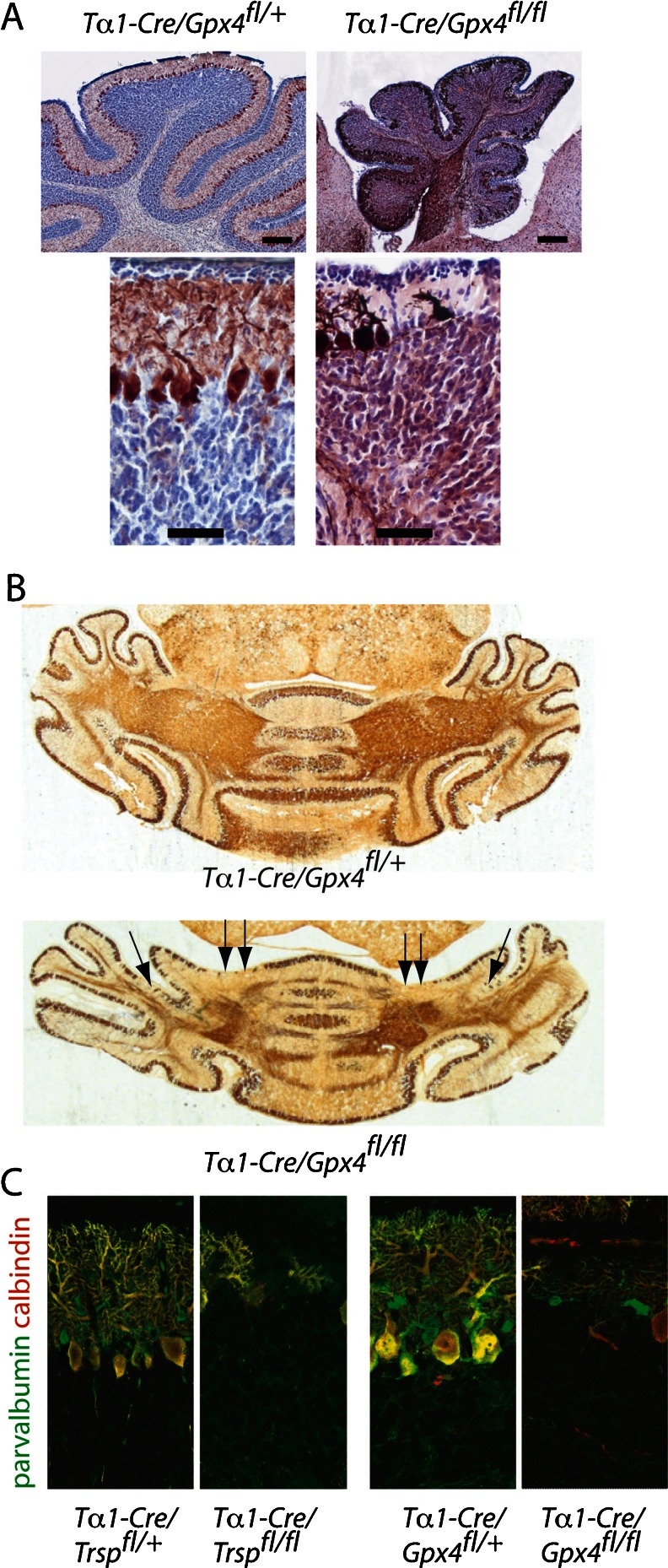
Conditional inactivation of *Gpx4* mimics *Trsp* deficiency. **a** Cerebellar hypoplasia and Purkinje cell loss in *Gpx4*-mutant mice on P12. Cresyl violet stain (*blue*) and α-calbindin (*brown*). *Scale bars* = 200 μm (*upper panel*) and 50 μm (*lower panel*). **b** Stripe-like loss (*arrows*) of Purkinje cells on P8. α-Calbindin (*brown*). **c** Few parvalbumin + cerebellar interneurons remain in *Gpx4*-mutant mice on P12. Purkinje cells are labeled *yellow* (parvalbumin is *green*, and calbindin is *red*), while interneurons express only parvalbumin (*green*)

The strong cerebellar phenotype of *T*α*1-Cre*/*Trsp* ^*fl/fl*^ mice directly prompted the question whether a single selenoprotein could be identified which is responsible for the observed cell death and impaired cell proliferation. We therefore crossed *T*α*1-Cre* with conditional glutathione peroxidase 4 (*Gpx4* ^*fl/fl*^) mice. These mice showed the same growth phenotype as *T*α*1-Cre*/*Trsp* ^*fl/fl*^ mice and did not survive beyond P13. More interestingly, *T*α*1-Cre*/*Gpx4* ^*fl/fl*^ mice phenocopied the cerebellar phenotype with respect to cerebellar hypoplasia and stunted growth of Purkinje cell dendrites or Purkinje cell loss (Fig. 3a). Purkinje cell loss again occurred along parasagittal stripes in *T*α*1-Cre*/*Gpx4* ^*fl/fl*^ mice (Fig. 3b). Since parvalbumin-positive GABAergic interneurons were the most affected cortical neurons, we asked whether interneurons in the cerebellum are also affected in our mutants. Purkinje cells (which are also GABAergic) express both calbindin and parvalbumin. Co-labeling of calbindin (red) and parvalbumin (green) in fluorescence microscopy therefore allows visualizing parvalbumin-positive interneurons (green) on a yellow background of Purkinje cells. As shown in Fig. 3c, parvalbumin-positive interneurons, presumably basket and/or stellate cells, are completely absent in *T*α*1-Cre*/*Trsp* ^*fl/fl*^ mice, while they partly remain in *T*α*1-Cre*/*Gpx4* ^*fl/fl*^ mice.

### Bergmann Glia

**Fig. 4:**
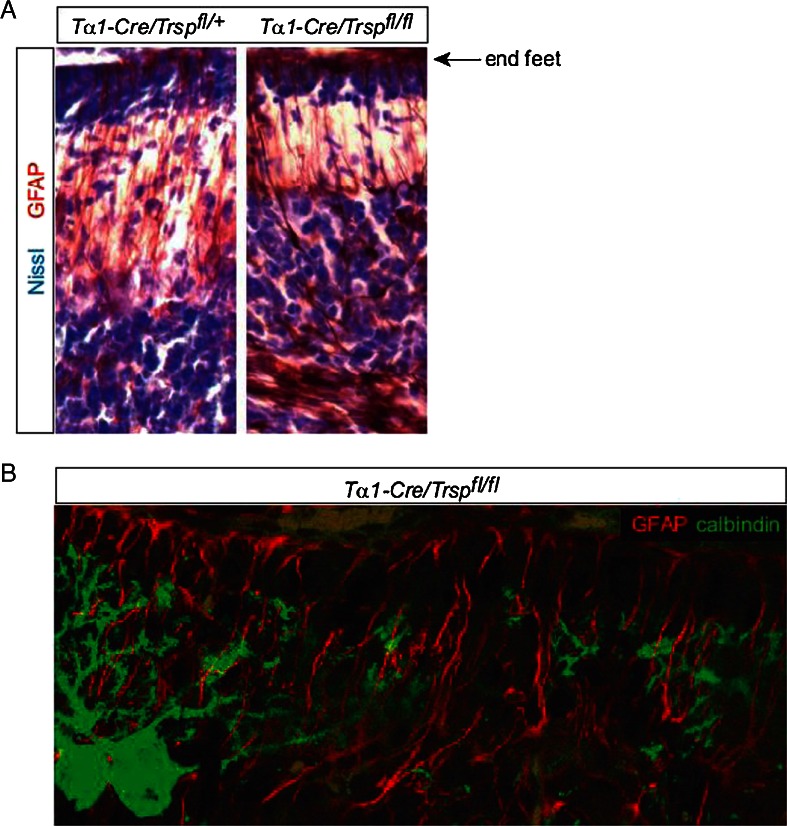
The density and orientation of radial glial fibers are not disrupted in *Trsp*-mutant cerebella on P12. **a** Staining of radial glia with an antibody against GFAP reveals intact pial contacts of radial glial fibers. **b** Double immunohistochemistry of calbindin and GFAP reveals that the radial glial network is not disrupted at sites of Purkinje cell loss

Staining of radial glia with GFAP as a marker did not reveal a reduction of Bergmann fiber density or radial glia morphology. Even in areas where Purkinje cell dendrites have degenerated, there is no indication of structural changes of Bergmann glial fibers (Fig. 4).

## Discussion

Initial observations in *SePP* ^*−/−*^ mice and later in *Apoer2* ^*−/−*^ mice established that selenoprotein expression in the mammalian brain is essential [4–7, 23–25]. Among the neurological phenotypes of affected mice were epilepsy/hyperexcitability and movement disorders. However, initial studies of *SePP* ^*−/−*^ mice did not demonstrate a clear pathology of the cerebellum, despite an abnormal gait of the mice [21, 23, 24]. In order to elicit the maximal phenotype associated with a lack of selenoprotein expression, we abrogated selenoprotein biosynthesis by inactivating *Trsp*, specifically in neurons using pan-neuronal Tα1-Cre (including cerebellum) and CamK-Cre (forebrain-specific, excluding cerebellum). In the cerebral cortex and hippocampus, parvalbumin-expressing interneurons did not develop at all, while parvalbumin-expressing cells in the globus pallidus were still detectable [12]. Most importantly, neuron-specific *CamK-Cre*/*Trsp* ^*fl/fl*^ mice exhibited hyperexcitability. Here, we describe that *T*α*1-Cre*/*Trsp* ^*fl/fl*^ mice, in which cerebellar neurons are affected, show cerebellar hypoplasia. Epilepsy and cerebellar hypoplasia are among the phenotypes of children afflicted with mutations in PCCA due to mutations in the *SEPSECS* gene [16]. Therefore, the essential role of selenoproteins in the mammalian brain can now be considered firmly established.

What could be the specific roles of selenoproteins in the brain? We show here that inactivation of Gpx4 alone already mimics a loss of all selenoproteins when cerebellar hypoplasia and Purkinje cell death are chosen as parameters. This finding is reminiscent of findings in forebrain interneurons and cultivated cortical neurons (mostly glutamatergic neurons) [12]. However, when comparing *T*α*1-Cre*/*Trsp* ^*fl/fl*^ mice and *T*α*1-Cre*/*Gpx4* ^*fl/fl*^ mice, it was noticed that loss of Gpx4 yields a slightly milder phenotype, indicating that besides Gpx4, a yet to be identified selenoprotein may contribute to the more severe pathology as observed in *T*α*1-Cre*/*Trsp* ^*fl/fl*^ mice.

Thioredoxin reductases are essential selenoproteins, but their inactivation in neurons using Tα1-Cre was shown not to cause to a cerebellar phenotype [15].

Cerebellar development represents a special paradigm since a secondary proliferating neuroepithelium is established in the course of its development. The first neurons to develop in the primary neuroepithelium of the cerebellar primordium are principal neurons of the deep nuclei and, most importantly, the Purkinje cells [26]. In a second step, neuronal precursors migrate through the Purkinje cell layer and form the external germinal layer where the precursors proliferate postnatally. The newly developed neurons, granule cells, then migrate along Bergmann glia processes inward through the Purkinje cell layer and establish the internal granule cell layer. Cerebellar hypoplasia is usually associated with a failure of the secondary neuroepithelium to produce the huge numbers of granule cells, which make up most of the cerebellar volume. Finally, GABAergic interneuron precursors are born in the primary neuroepithelium and migrate outwards where basket cells mature close to Purkinje cells and stellate cells migrate into the molecular layer and reside among the Purkinje cell dendrites. In our *T*α*1-Cre*/*Trsp* ^*fl/fl*^ mouse model, Purkinje cell death was observed at a stage when the Purkinje cell layer is already formed; hence, we find gaps in the layer. The *T*α*1-Cre* transgene is only expressed in neurons, not neuronal precursors consistent with the loss of postmitotic Purkinje cells. Why Purkinje cell degeneration occurs often along parasagittal stripes (e.g., preferentially in aldolase C-negative cells in our model) is not known but may be associated with the functional patterning of the cerebellum [22]. Purkinje cells secrete along their dendrite factors stimulating granule cell proliferation, including sonic hedgehog and others [27]. A reduced number of Purkinje cells may thus entail reduced promitotic stimulation of granule cell precursors in the external germinal layer. Migration of granule cells along Bergmann fibers may occur normally, since we did not find evidence of radial glia defects. Targeting neural precursors and Bergmann fibers, *nestin-Cre; Txnrd1* ^*fl/fl*^ mice also suffer cerebellar hypoplasia associated with defective foliation/migration disorganized Bergmann fibers [15]. This may bear some relevance in the case of global selenoprotein deficiency as in PCCA. Of note, our *T*α*1-Cre* transgene targets only postmitotic neurons and not glia. We find, however, TUNEL-positive cells mostly in the internal granule cell layer, suggesting that death of granule cells contributes to the observed cerebellar hypoplasia. Whether granule cells degenerate in a cell autonomous process or because their synaptic targets, Purkinje cells, degenerate is not known. We observed that GABAergic interneurons, which express parvalbumin once they reach their destination in the cerebellar cortex, are virtually absent in *T*α*1-Cre*/*Trsp* ^*fl/fl*^ mice. Their numbers are also reduced in Gpx4 mutants, but occasionally, we observed few of these neurons in the Purkinje cell layer.

It is interesting to note that the neurons most severely affected by the lack of selenoprotein expression (or Gpx4 alone) are GABAergic neurons that also express the calcium-binding protein parvalbumin: cortical interneurons, cerebellar interneurons, and Purkinje cells. Cortical GABAergic neurons expressing calbindin or calretinin are less affected [12]. However, neurons in the globus pallidus are also GABAergic and express parvalbumin and are not affected, or at least, they do not degenerate up to the latest time points we have studied them. Therefore, further studies are needed to define the mechanism of how neurons are damaged by the lack of selenoproteins. While “oxidative stress” is often invoked and massive oxidation of cell constituents is assumed, we speculate that specific developmental mechanisms are targeted. Dysregulations of redox-sensitive pathways are our prime suspects for further study, because oxidative stress may not be very cell-specific at all. For example, signaling by receptor tyrosine kinases often entails transient oxidation of protein phosphatases. It has been shown that lipid hydroperoxides generated enzymatically by 12/15-lipoxygenase efficiently inactivate kinases [28]. Targeting of Gpx4, a lipid hydroperoxide-specific peroxidase, recapitulates the cerebellar phenotype, and we speculate that selenoprotein deficiency may act along such a mechanism.
